# Repeatability and reproducibility of human brain morphometry using three‐dimensional magnetic resonance fingerprinting

**DOI:** 10.1002/hbm.25232

**Published:** 2020-10-22

**Authors:** Shohei Fujita, Guido Buonincontri, Matteo Cencini, Issei Fukunaga, Naoyuki Takei, Rolf F. Schulte, Akifumi Hagiwara, Wataru Uchida, Masaaki Hori, Koji Kamagata, Osamu Abe, Shigeki Aoki

**Affiliations:** ^1^ Department of Radiology Juntendo University Tokyo Japan; ^2^ Department of Radiology The University of Tokyo Tokyo Japan; ^3^ Imago7 Foundation Pisa Italy; ^4^ IRCCS Stella Maris Pisa Italy; ^5^ MR Applications and Workflow GE Healthcare Tokyo Japan; ^6^ GE Healthcare Munich Germany; ^7^ Department of Radiological Sciences Tokyo Metropolitan University Tokyo Japan; ^8^ Department of Radiology Toho University Omori Medical Center Tokyo Japan

**Keywords:** human brain imaging, magnetic resonance fingerprinting, morphometry, relaxometry, repeatability, reproducibility

## Abstract

Three‐dimensional (3D) Magnetic resonance fingerprinting (MRF) permits whole‐brain volumetric quantification of T1 and T2 relaxation values, potentially replacing conventional T1‐weighted structural imaging for common brain imaging analysis. The aim of this study was to evaluate the repeatability and reproducibility of 3D MRF in evaluating brain cortical thickness and subcortical volumetric analysis in healthy volunteers using conventional 3D T1‐weighted images as a reference standard. Scan‐rescan tests of both 3D MRF and conventional 3D fast spoiled gradient recalled echo (FSPGR) were performed. For each sequence, the regional cortical thickness and volume of the subcortical structures were measured using standard automatic brain segmentation software. Repeatability and reproducibility were assessed using the within‐subject coefficient of variation (wCV), intraclass correlation coefficient (ICC), and mean percent difference and ICC, respectively. The wCV and ICC of cortical thickness were similar across all regions with both 3D MRF and FSPGR. The percent relative difference in cortical thickness between 3D MRF and FSPGR across all regions was 8.0 ± 3.2%. The wCV and ICC of the volume of subcortical structures across all structures were similar between 3D MRF and FSPGR. The percent relative difference in the volume of subcortical structures between 3D MRF and FSPGR across all structures was 7.1 ± 3.6%. 3D MRF measurements of human brain cortical thickness and subcortical volumes are highly repeatable, and consistent with measurements taken on conventional 3D T1‐weighted images. A slight, consistent bias was evident between the two, and thus careful attention is required when combining data from MRF and conventional acquisitions.

## Graphical abstract

Three‐dimensional (3D) Magnetic resonance fingerprinting (MRF) permits whole‐brain volumetric quantification of T1 and T2 relaxation values, potentially replacing conventional T1‐weighted structural imaging for common brain imaging analysis. The aim of this study was to evaluate the repeatability and reproducibility of 3D MRF in evaluating brain cortical thickness and subcortical volumetric analysis. 3D MRF measurements of human brain cortical thickness and subcortical volumes are highly repeatable, and consistent with measurements taken on conventional 3D T1‐weighted images.

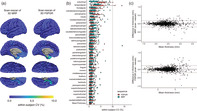

## 1 INTRODUCTION

Brain morphological information and tissue relaxation time are important pieces of biological information that can be acquired by magnetic resonance imaging (MRI). Changes in brain morphometry, including regional cortical thickness and subcortical volumes, are reportedly associated with brain development, aging, and a variety of psychiatric and neurological disorders (Fotenos, Snyder, Girton, Morris, & Buckner, 2005). These morphological metrics can quantify neuroanatomical features, and accurate measurements could enable better understanding of individual clinical, behavioral, and genetic characteristics (Sabuncu et al., 2016). Relaxation time is a quantitative tissue property that allows objective tissue characterization and is reportedly sensitive to underlying disease‐induced changes. T1 and T2 relaxation times are altered in specific patterns in neurological diseases such as Alzheimer's disease (Dean 3rd et al., 2017; House, St Pierre, Foster, Martins, & Clarnette, 2006), multiple sclerosis (Blystad et al., 2016; Hagiwara et al., 2019), and epilepsy (Townsend, Bernasconi, Pike, & Bernasconi, 2004; von Oertzen et al., 2002). Moreover, a combined analysis of morphology and relaxometry has been applied to tasks including automatic detection and quantification of lesions and local brain regions (Pell, Briellmann, Waites, Abbott, & Jackson, 2004; Specht, Minnerop, Muller‐Hubenthal, & Klockgether, 2005). Morphological information and relaxation times can provide complementary information (Bernasconi et al., 2000; Fujita et al., 2019; Knight, Wearn, Coulthard, & Kauppinen, 2019), potentially allowing for a more robust and comprehensive tissue evaluation.

Magnetic resonance fingerprinting (MRF) is a multiparametric relaxometry technique that permits simultaneous acquisition of MR properties with a single, efficient acquisition (Ma et al., 2013). MRF depends on a pattern‐matching approach in which time‐dependent signals characterizing various relaxation processes unique to each tissue are matched to a dictionary of theoretically simulated signals (Bipin Mehta et al., 2019). Previous in vitro and in vivo studies demonstrated high reproducibility and repeatability of T1 and T2 values obtained with MRF (Buonincontri et al., 2019; Jiang et al., 2017; Kato et al., 2019; Korzdorfer et al., 2019). The MRF framework enables the acquisition of various quantitative metrics including, but not limited to, T1 and T2 values. Promising results have been reported in a wide range of clinical imaging applications, including the brain (Badve et al., 2017), liver (Chen et al., 2016), prostate (Panda et al., 2019; Panda et al., 2019), heart (Liu, Hamilton, Rajagopalan, & Seiberlich, 2018), and breasts (Chen et al., 2019; Panda et al., 2019).

Recent efforts have been made to extend the volumetric coverage of MRF acquisition. Several three‐dimensional (3D) MRF approaches have been proposed which enable whole‐brain volumetric quantification of T1 and T2 relaxation times in a clinically applicable timeframe (Cao et al., 2019; Liao et al., 2017; Ma et al., 2018). These approaches provide high‐resolution 3D structural data which could be generated from the acquired 3D T1 and T2 maps, making the approach more feasible in clinical practice. These high‐resolution 3D quantitative maps by MRF may replace conventional structural data for common brain imaging analysis and in the clinics where a T1‐weighted image is required. However, studies focusing on the reliability of brain morphometry derived from MRF have been limited, despite its clinical relevance.

The purpose of this study was to evaluate the scan‐rescan repeatability of 3D MRF‐derived measurements, and to evaluate the reproducibility of 3D MRF‐derived morphological measurements using conventional 3D T1‐weighted structural imaging‐derived measurements as reference values.

## 2 METHODS

### 2.1 MRI acquisitions

Data were collected with the approval of the Institutional Review Board, and all subjects provided written informed consent prior to the scan. This study included 21 healthy volunteers (12 women and 9 men; mean age, 41.3 ± 14.6; age range, 22–72 years). All participants had a negative history of major neurological, psychiatric, or cognitive impairments. Scans were performed using a 3T scanner (Discovery 750 w, GE Healthcare, Waukesha, WI) with a 32‐channel head coil. Scan‐rescan tests of both 3D MRF and 3D T1‐weighted fast spoiled gradient recalled echo (FSPGR) sequences were performed for all participants. The subjects were repositioned between the scan and rescan tests. The MRF data acquisition and reconstruction was performed as described by Gómez et al (Gómez et al., 2020). Briefly, the 3D MRF sequence was based on steady‐state free precession (SSFP) with a 3D spiral projection trajectory. The acquisition schedule consisted of a series of variable flip angle hard pulses preceded by an adiabatic inversion pulse to encode for T1 and T2. The flip angle pattern consisted of a triangularly shaped increasing linear ramp, followed by a decreasing linear ramp and a constant section (Figure S1). Repetition time (TR) and echo time (TE) were kept constant during the whole experiment, respectively to avoid off‐resonance dependency (Cencini et al., 2019) and to achieve minimal acquisition time. The whole schedule was repeated to increase k‐space sampling. Each frame was acquired with a single variable‐density spiral interleave, followed by a spoiling gradient along the z‐axis at the end of the TR, achieving 4π dephasing per pixel. The spiral interleave was rotated in‐plane by linear increments within each TR, while the spiral plane underwent golden‐angle rotation within each schedule repetition to achieve 3D k‐space coverage.

Reconstruction was performed by processing under‐sampled data with k‐space‐weighted view‐sharing for anti‐aliasing. First, sampling for each frame was increased by sharing data from the neighboring 880 frames (corresponding to the schedule length). The amount of sharing was inversely proportional to the trajectory sampling density. Then, k‐space data were projected on a temporal subspace obtained by Singular Value Decomposition (McGivney et al., 2014) of the MRF dictionary. After subspace projection, gridding with nonuniform fast Fourier transformation (FFT) and 3D FFT was used to obtain the 3D volume in the image space. Final images were generated with adaptive coil combination (Walsh, Gmitro, & Marcellin, 2000) after coil sensitivity estimation. Data from the first schedule repetition was excluded from the reconstruction due to the different spin dynamics with respect to the others. The acquisition parameters for MRF were as follows: TR, 12 ms; TE, 0.5 ms; field‐of‐view (FOV), 200 mm × 200 mm × 200 mm; matrix size, 200 mm × 200 mm × 200 mm; and acquisition time, 9 min 51 s.

Conventional 3D T1‐weighted structural images were acquired for calculating reference brain morphology metrics. FSPGR imaging parameters were sagittal acquisition; TR/TE/inversion time (TI) (7.7/3.1/400 ms) FOV (256 mm × 256 mm); matrix size (256 mm × 256 mm); section thickness (1.0 mm); flip angle (11°); receiver bandwidth (244.1 Hz/pixel); and acquisition time (5 min 45 s). FSPGR raw data were reconstructed using the standard vendor reconstruction pipeline, including the application of a radial Fermi apodization window in the k‐space, Cartesian Fast Fourier Transform, and Sum‐Of‐Squares channel combination. To eliminate the effect of voxel size differences in the morphology metrics, the isotropic MRF spatial resolution was set at 1.0 mm and matched with that of FSPGR imaging. Based on the Alzheimer's Disease Neuroimaging Initiative (ADNI) study (Jack Jr. et al., 2008), 1.0 mm isotropic data was chosen. MRF and FSPGR images exhibiting common artifacts, such as ringing, blurring, and ghosting, were excluded. One participant was excluded because of the presentation of motion artifacts in both 3D MRF and FSPGR.

### 2.2 Data processing

A dictionary was generated using the extended phase graph formalism (EPG), with T1 values ranging from 10 to 100 ms, with 10 ms intervals; 100 ms to 1 s, with 20 ms intervals; 1 to 2 s, with 50 ms intervals; and 2 to 6 s, with 100 ms intervals, and T2 values ranging from 2 to 100 ms, with 2 ms intervals; 100 to 150 ms, with 5 ms intervals; 160 to 300 ms, with 10 ms intervals; 300 to 800 ms, with 50 ms intervals; 800 to 1,600 ms, with 100 ms; and 1,600 to 3,000 ms, with 200 ms intervals (Naganawa et al., 2019). To account for the effect on spin dynamics of schedule repetition, the EPG simulation was repeated two times, using the magnetization state at the end of the first iteration as an input for the second. The dictionary was obtained by retaining the results of the second iteration. As shown in Figure S2, after the second repetition the magnetization at the beginning of the schedule reaches a pseudo‐steady state, avoiding the necessity of further iterations of the algorithms to obtain the signal evolution. This also justifies the choice of discarding only the data from the first schedule repetition during the image reconstruction step.

The MRF T1 and T2 maps were obtained by performing a maximum inner product search (Ma et al., 2013). MRF‐derived T1 maps were postprocessed using voxels and the following formula to generate synthetic T1‐weighted images:

$$S=1-2e^{-\mathrm{TI}/T1}$$

S is the output signal, T1 is the T1 value obtained by MRF, and TI is the inversion time, virtually set to 1,300 ms. Inspired by MPRAGE‐based morphometry (O'Brien et al., 2014), proton density was not included in the synthetic T1‐weighted generation to avoid the need of a bias field correction step. We performed this redundant process because the automatic brain segmentation software only accepts input of T1‐weighted contrast images.

### 2.3 Brain segmentation

Each T1‐weighted structural image was analyzed using automatic brain segmentation software. Individual and specific regional cortical thickness defined in the Desikan–Killiany Atlas (Desikan et al., 2006), was obtained using FreeSurfer (Version 6.0) (Dale, Fischl, & Sereno, 1999; Fischl, 2012) with the default *recon‐all* command. The subcortical structural volumes (Patenaude, Smith, Kennedy, & Jenkinson, 2011) were obtained with the default *run_first_all* command in the FMRIB integrated registration and segmentation tool (FIRST), implemented in the FMRIB Software Library v. 5.0.9 in addition to FreeSurfer, because of the high variability reported in subcortical gray matter segmentation when using FreeSurfer (Dale et al., 1999). The segmented masks were applied to the original T1 and T2 maps to calculate the mean T1 and T2 values for each anatomical region. Because the quantitative maps are inherently aligned with the T1‐weighted images, no additional registration steps were required for alignment with the masks. Bilateral regional values were averaged for subsequent analysis. No manual corrections were performed because the aim of the study was to evaluate whether the maps obtained with 3D MRF could be reliably used for automated volumetric analysis.

### 2.4 Statistical analyses

R program v. 3.3.0 (R Core Team [2016]. R: A language and environment for statistical computing. R Foundation for Statistical Computing, Vienna, Austria. URL https:// www.R-project.org/) was used for all statistical analyses, and figures were produced using the ggplot2 package (Wickham, 2016). Scan–rescan repeatability was assessed by the within‐subject coefficient of variation (wCV) and intraclass correlation coefficient (ICC) for each anatomical volume/thickness. We would consider MRF repeatable if the wCV was low and the ICC was high. Bland–Altman plots were generated to assess the agreement between repeated measurements across brain regions. The limits of agreement (LOA) were defined as the mean ± 1.96 × *SD* of the difference between the scan and rescan values. Reproducibility between the 3D MRF and FSPGR sequence‐derived measurements was assessed by calculating the percent relative difference and ICC for each anatomical volume/thickness. We would consider MRF reproducible if the percent relative difference was low and the ICC was high. The ICC estimates of agreement were categorized as follows: poor (0.01–0.39), fair (0.40–0.59), good (0.60–0.74), and excellent (0.75–1.00) (Cicchetti, 1994).

## 3 RESULTS

**FIGURE 1:**
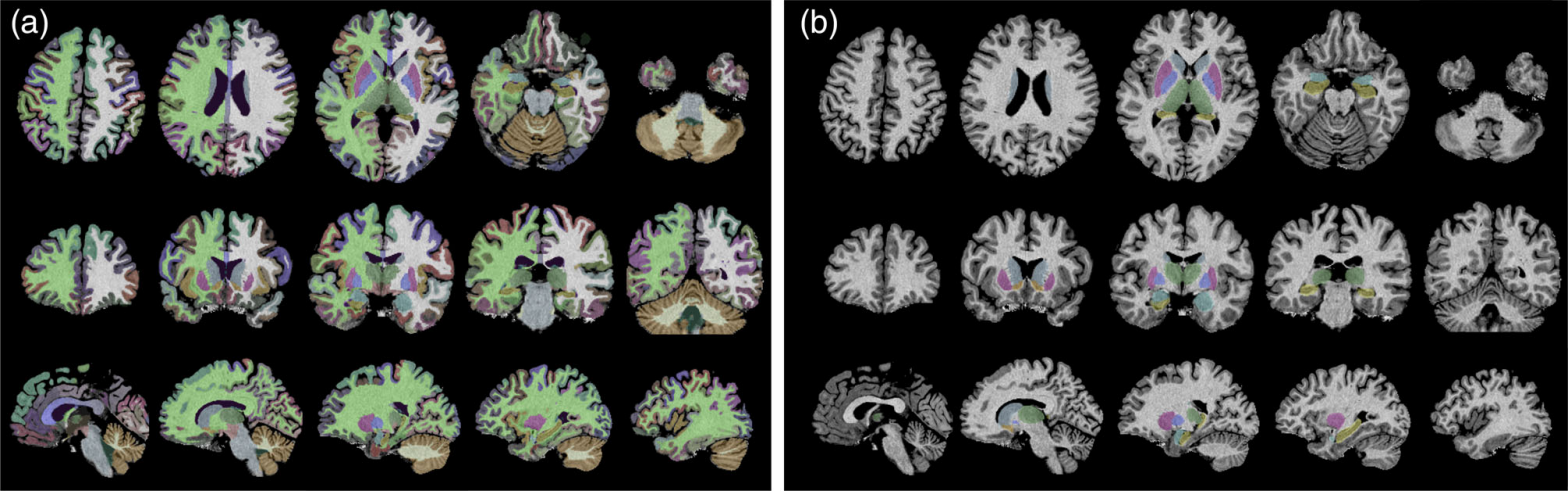
Representative (a) FreeSurfer and (b) FMRIB Integrated Registration and Segmentation Tool (FIRST) labels created from automated segmentation of brain regions using 3D magnetic resonance fingerprinting (MRF)‐based T1‐weighted images. Segmentation results are overlaid with the 3D MRF‐based T1‐weighted images

Twenty participants were included in the final analysis (11 women and 9 men; mean age, 41.7 ± 14.8; age range, 22–72 years). Representative 3D MRF sequence‐derived T1‐weighted images obtained with FreeSurfer and FIRST outputs are shown in Figure 1.

### 3.1 Measurement of cortical thickness

**FIGURE 2:**
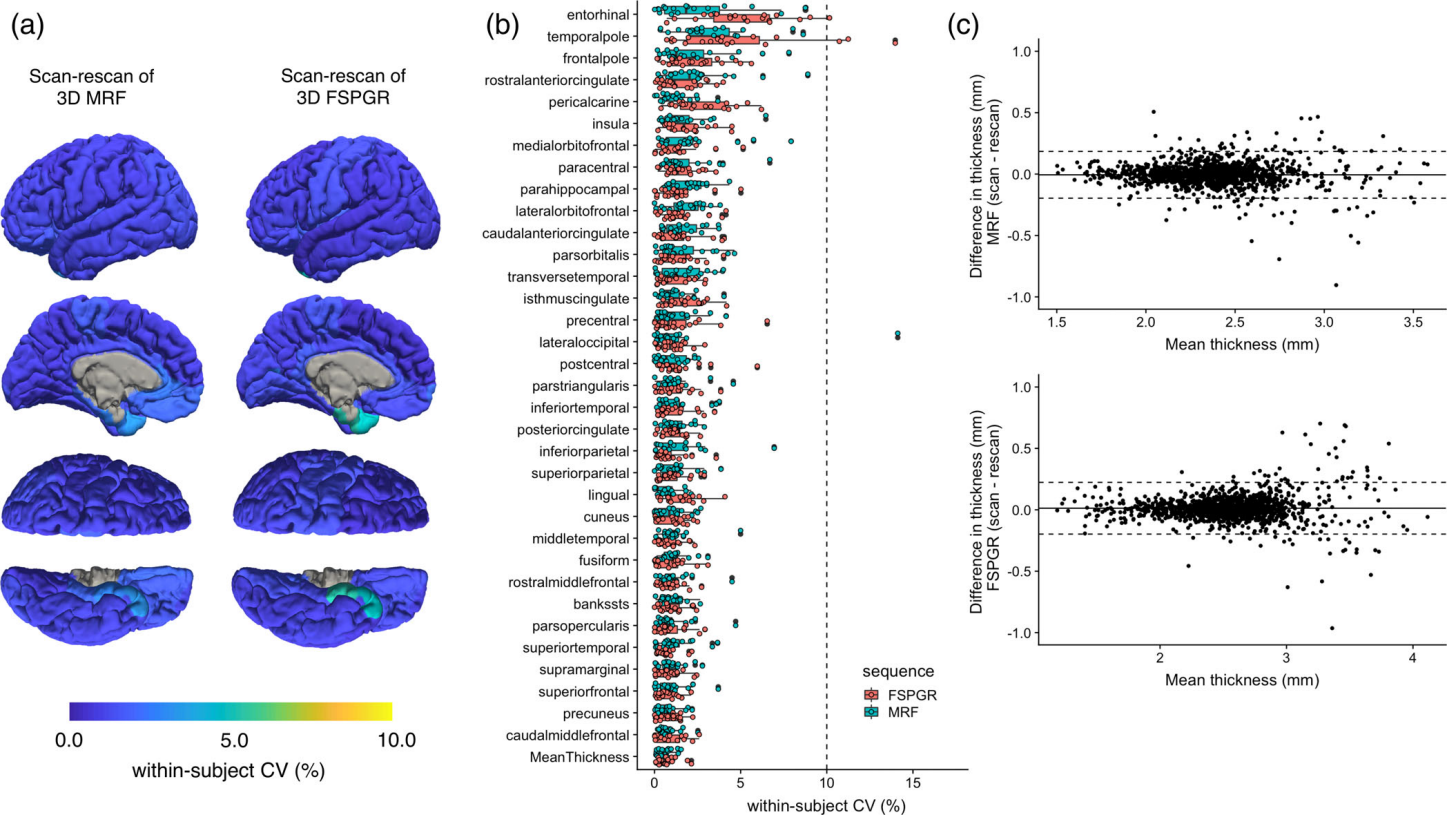
Scan‐rescan repeatability of cortical thickness for 3D MRF and 3D FSPGR. (a) Scan‐rescan within‐subject coefficient of variation (wCV) among scans are overlaid on an inflated brain surface for 3D MRF and 3D FSPGR. (b) Scan‐rescan wCV of cortical thickness for 3D MRF and FSPGR (Boxes indicate the interquartile range [25–75%] and circles indicate sample data points). (c) Bland–Altman plots of the scan‐rescan variation of cortical thickness across all subjects in all regions using (top) 3D MRF and (bottom) 3D FSPGR

The wCV of cortical thickness was similar across all regions between 3D MRF (1.4 ± 0.6%) and FSPGR (1.4 ± 1.0%) (Figure 2). Cortical thicknesses corresponding to all regions showed less than 5% wCV in both 3D MRF and FSPGR, except the entorhinal cortical thickness obtained using FSPGR. The mean ICC values across all regions were 0.88 and 0.94 for 3D MRF and FSPGR, respectively (Table S1). The Bland–Altman plots for scan‐rescan repeatability of local cortical thickness in all regions are shown in Figure 2c. The upper and lower LOA values were 0.18 and − 0.20 mm for 3D MRF and 0.22 and − 0.20 mm for FSPGR. Both methods demonstrated excellent repeatability.

**FIGURE 3:**
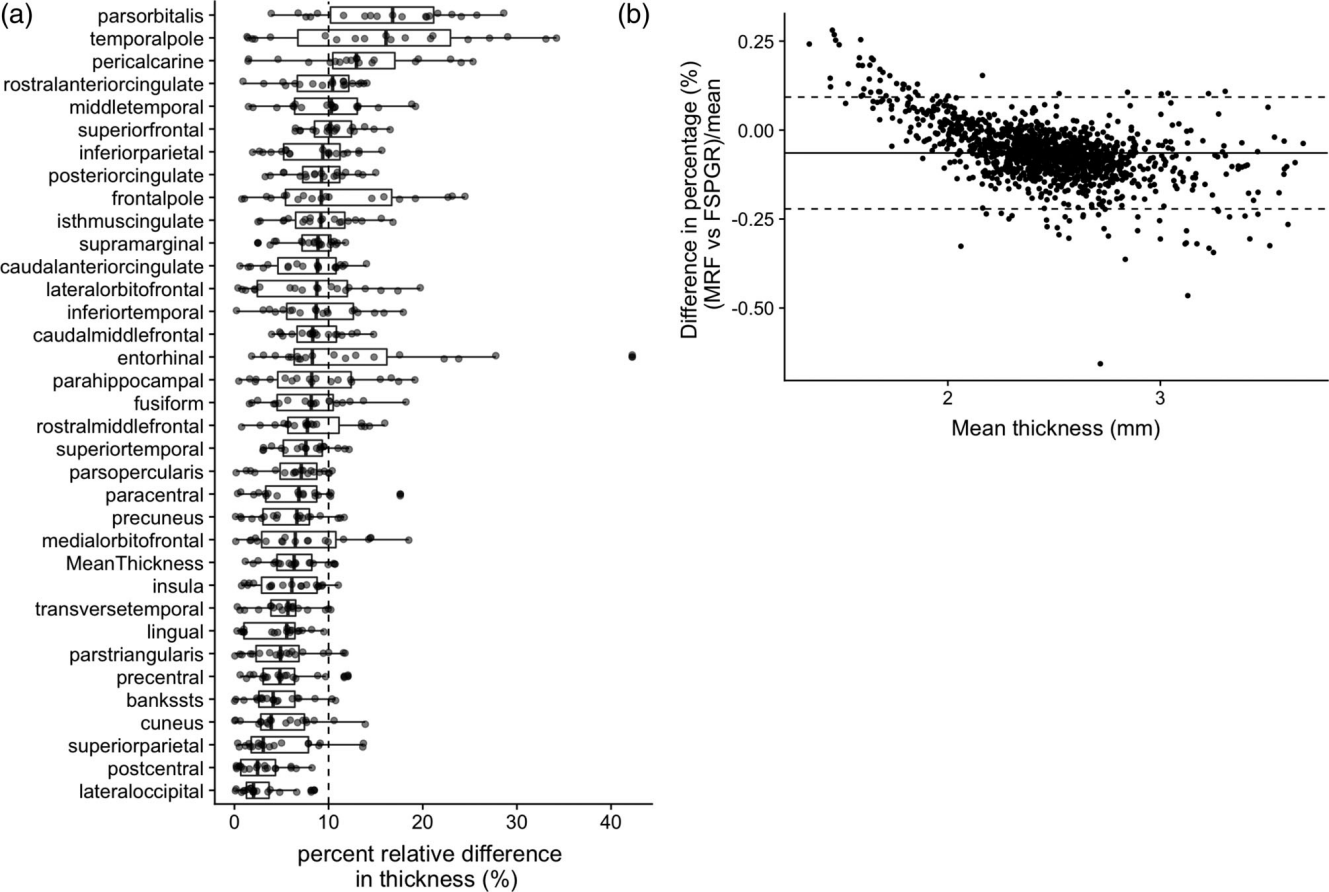
Difference between MRF‐ and FSPGR‐derived cortical thickness. (a) Percent relative difference between MRF‐ and FSPGR‐derived cortical thickness (Boxes indicate the interquartile range [25–75%] and circles indicate sample data points). (b) Bland–Altman plots of showing bias of cortical thickness across all subjects in all regions using 3D MRF and 3D FSPGR. Slight bias was observed between MRF and FSPGR‐derived cortical thicknesses in areas with relatively thin cortical thickness (see Figure S3 for detail)

**FIGURE 4:**
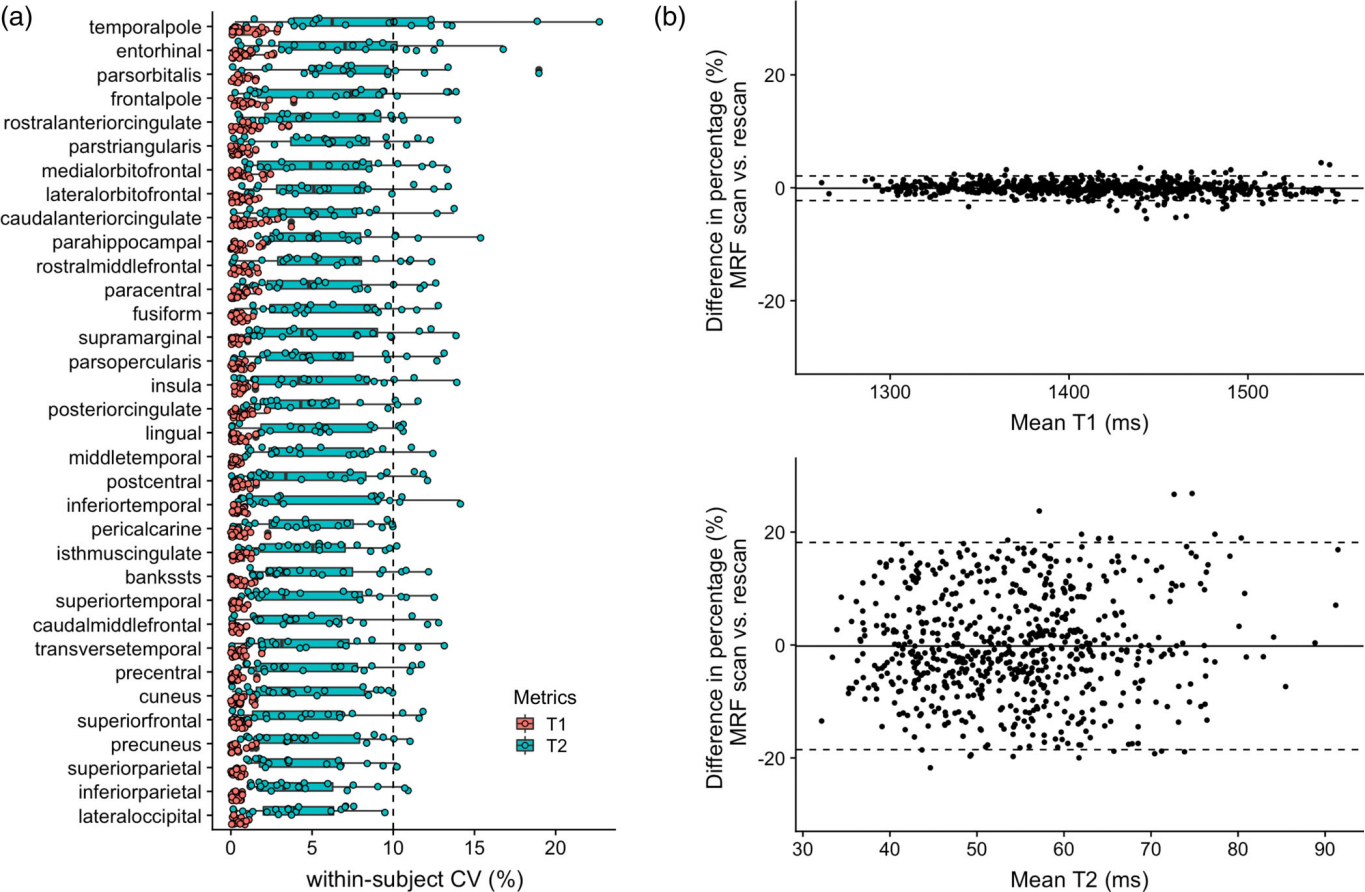
Scan‐rescan repeatability of T1 and T2 value of cortices derived with 3D MRF. (a) Scan‐rescan within‐subject coefficient of variation (wCV) of 3D MRF‐derived T1 and T2 values (Boxes indicate the interquartile range [25–75%] and circles indicate sample data points). (b) Bland–Altman plots of scan‐rescan variation in 3D MRF‐derived (top) T1 and (bottom) T2 values of cortices across all subjects

The percent relative difference between 3D MRF and FSPGR in cortical thickness across all regions was 8.0 ± 3.2%, indicating good reproducibility (Figure 3). Overall, MRF‐derived thicknesses were slightly thinner (0.17 mm) than that of FSPGR. Noticeable bias between the two, were observed in the cortex areas with thin cortical thickness (Figure 3b), especially in the primary visual cortex area (Figure S3). The ICC across all regions was 0.67 ± 0.21, and 62% of the regional areas showed good to excellent agreement (Table S1). The mean whole brain cortical thicknesses determined using 3D MRF and 3D FSPGR were 2.3 ± 0.1 mm and 2.5 ± 0.1 mm, respectively. The T1 and T2 values obtained by MRF of the same cortical regions varied by 0.3–1.1% and by 3.9–8.2%, respectively (Figure 4).

### 3.2 Volumetry of subcortical structures

**FIGURE 5:**
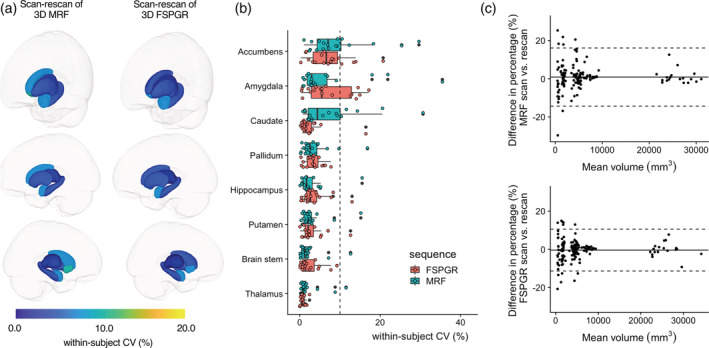
Scan‐rescan repeatability of subcortical volumes for 3D MRF and 3D FSPGR. (a) Scan‐rescan within‐subject coefficient of variation (wCV) among scans is overlaid on an inflated brain surface for 3D MRF and 3D FSPGR. (b) Scan‐rescan wCV of subcortical volumes for 3D MRF and FSPGR (Boxes indicate the interquartile range [25–75%] and circles indicate sample data points). (c) Bland–Altman plots of the scan‐rescan variation in subcortical structure volumes across all subjects in all structures using (top) 3D MRF and (bottom) 3D FSPGR

The wCV of the subcortical volume was 2.9 ± 2.0% with 3D MRF and 3.0 ± 2.1% with FSPGR using FIRST (Figure 5). All structures showed less than 10% wCV in both 3D MRF and FSPGR. The mean ICC across all regions was 0.85 ± 0.10 and 0.90 ± 0.08 for 3D MRF and FSPGR, respectively (Table S1). All structures showed good to excellent agreement in both 3D MRF and FSPGR. The Bland–Altman plots showing scan‐rescan repeatability for local subcortical structure volumes in all regions are presented in Figure 5c. The upper and lower LOA values were 16 and − 14% with 3D MRF and 11 and − 11% for FSPGR. Volume of the nucleus accumbens in MRF showed relatively low scan‐rescan repeatability (wCV of 9.3%). The wCV of the subcortical volume was 1.6 ± 1.1% with 3D MRF and 1.9 ± 2.1% with FSPGR using FreeSurfer (Figure S4). The volume of the nucleus accumbens in FSPGR showed relatively low scan‐rescan repeatability, with wCV of 7.5%, using FreeSurfer.

**FIGURE 6:**
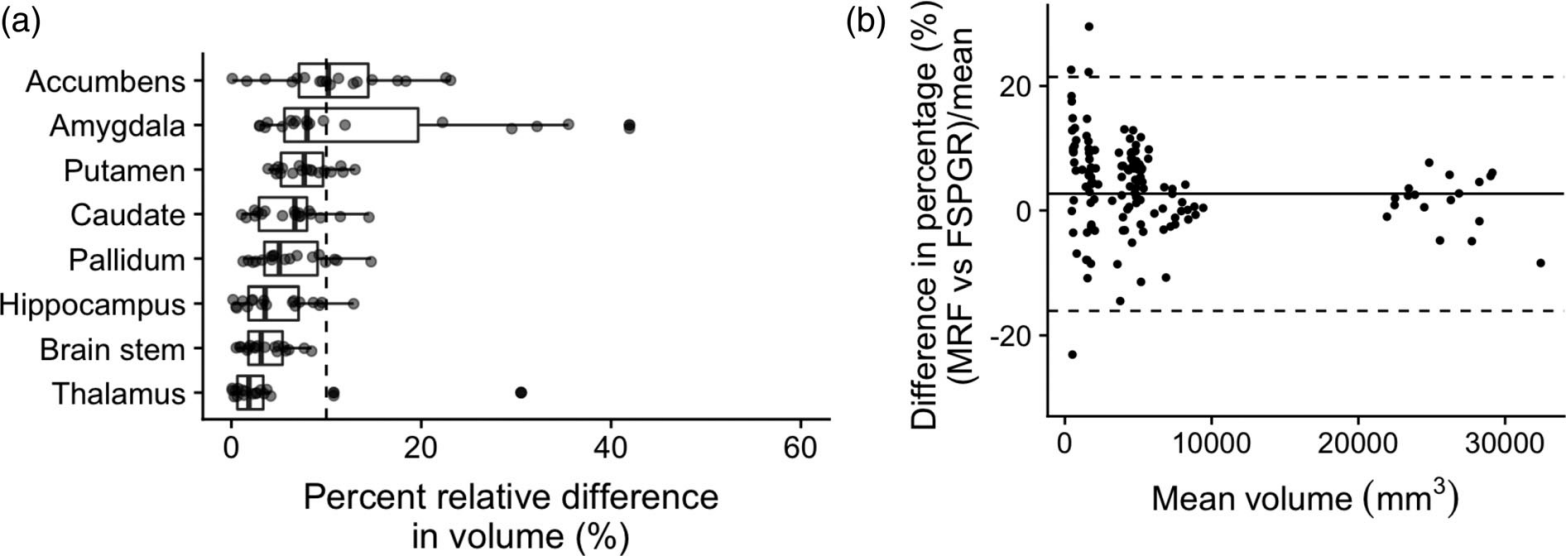
Difference between MRF‐ and FSPGR‐derived subcortical volumes. (a) Percent relative difference between MRF‐ and FSPGR‐derived subcortical volumes (Boxes indicate the interquartile range [25–75%] and circles indicate sample data points). (b) Bland–Altman plots of showing bias of subcortical volumes across all subjects in all structures using 3D MRF and 3D FSPGR

**FIGURE 7:**
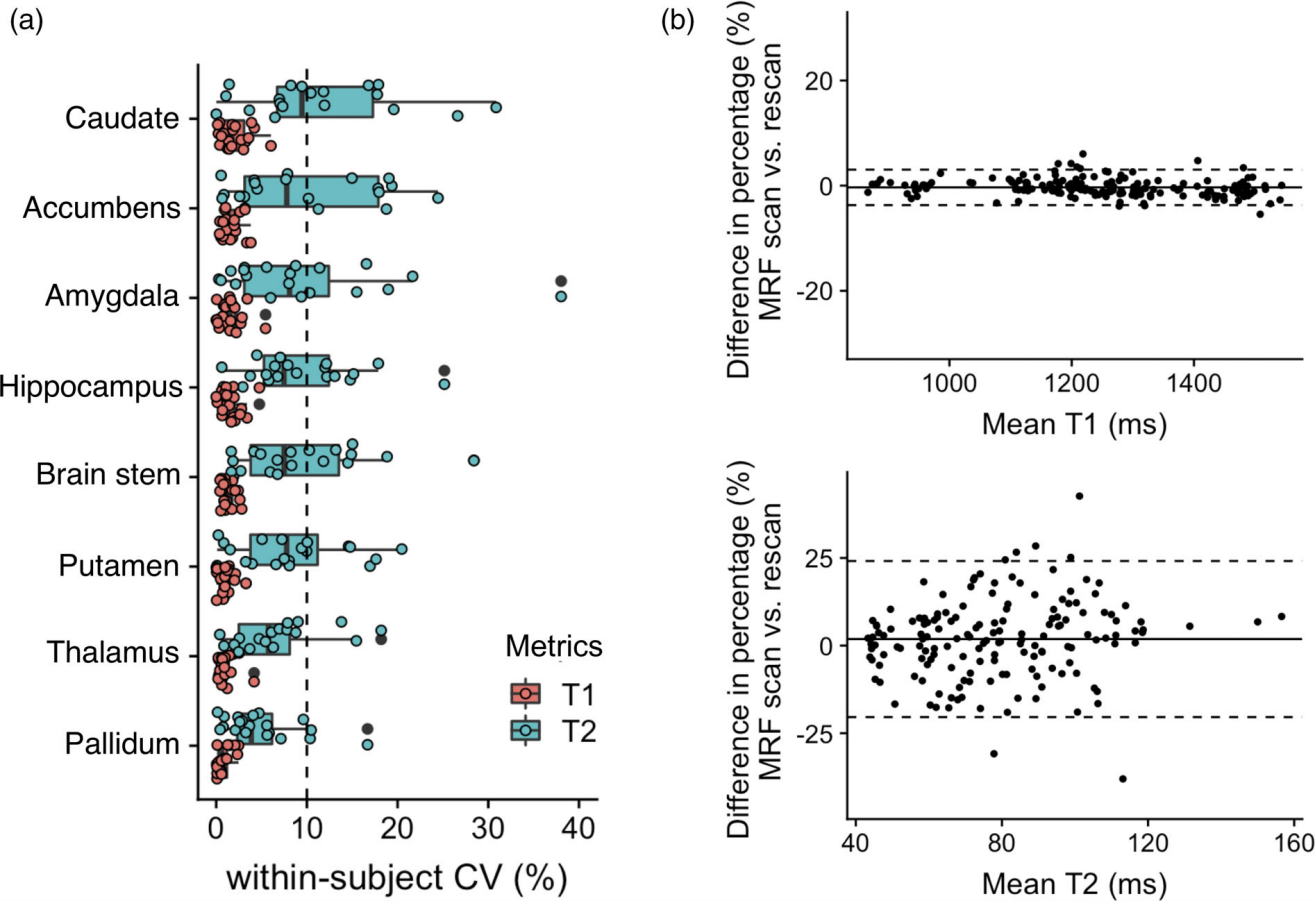
Scan‐rescan repeatability of T1 and T2 value of subcortical structures derived with 3D MRF. (a) Scan‐rescan within‐subject coefficient of variation (wCV) of 3D MRF‐derived T1 and T2 values (Boxes indicate the interquartile range [25–75%] and circles indicate sample data points). (b) Bland–Altman plots of scan‐rescan variation in 3D MRF‐derived (top) T1 and (bottom) T2 values of subcortical structures across all subjects

The percent relative difference between 3D MRF and FSPGR in subcortical structure volume across all regions was 7.1 ± 3.6%, indicating high reproducibility (Figure 6). No noticeable bias was observed between MRF‐derived volumes of subcortical structures and that of FSPGR (Figure S4). The ICC across all regions was 0.78 ± 0.15, and all regions except the amygdala showed good to excellent agreement (Table S1). The T1 and T2 values obtained by MRF of the same subcortical regions varied by 0.5–2.0% and by 3.6–12.9%, respectively (Figure 7 and Figure S5).

## 4 DISCUSSION

The evaluation of repeatability and reproducibility is essential for the effective use of quantitative metrics. The present study evaluated the in vivo scan‐rescan repeatability of MRF and evaluated the reproducibility by comparing the MRF‐derived measurements to that of conventional 3D T1‐weighted structural imaging. The findings of this study indicate that 3D MRF provides highly repeatable measurements of regional cortical thicknesses and subcortical volumes, and overall good agreement with conventional 3D T1‐weighted structural imaging‐derived measurements. To our knowledge, this is the first study to evaluate the repeatability and reproducibility of 3D MRF‐derived morphometry of the human brain.

The repeatability of 3D MRF‐derived metrics was comparable with that of FSPGR for cortical thickness and subcortical volumes. Relatively low repeatability was observed in the cortical thickness of the entorhinal region, temporal pole, and frontal pole and the volume of the nucleus accumbens, in both 3D MRF and FSPGR datasets. This result was consistent with those of previous studies, which reported relatively low reliability in these regions based on conventional 3D T1‐weighted imaging (Morey et al., 2010; Tustison et al., 2014). The cortical thickness estimated by 3D MRF tended to be thinner than that estimated by FSPGR, possibly due to the different flip angle and acquisition settings, resulting in different gray/white matter contrast. The range of cortical thicknesses determined using MRF were comparable to those reported by previous studies based on both postmortem and conventional T1‐weighted imaging studies (Desikan et al., 2006). Although the agreement between the measurements obtained by the two sequences were high, we found noticeable bias between the two in cortex areas with thin cortical thickness (Figure 3b), where MRF measurements were thicker than that of FSPGR. Figure S3 helps explain the reason, where FSPGR seemed to missegment in the primary visual areas. The FSPGR tend to have relatively low gray/white matter contrast compared to other T1‐weighted imaging, which has been explained by a previous study (Han et al., 2006). The contrast due to high degree of myelination, well known in the primary visual areas (Braitenberg & Schüz, 1998), has decreased the gray/white matter contrast resulting in missegmentation in FSPGR.

It should be emphasized that the variability of the segmentation does not depend exclusively on the acquisition sequence but also depends on the compatibility with the post‐processing software. We have performed subcortical structure segmentation using two segmentation software, FreeSurfer and FIRST, to evaluate the effects of software and that of the MRF sequence. Although the exact separation of the variability arising from the sequence and software is difficult, the results show that the MRF keeps wCV of volumes of subcortical structures less than 10% in either software, and thus, we expect the errors derived from the sequence scheme to be in single‐digit percentages, at most.

An inherent benefit of using MRF for morphometry is its perfect alignment with quantitative property maps. Because the acquisition is performed in a single scan, all the quantitative property maps directly correspond to the patient's anatomy. This property allows the direct use of segmentation results as the volume of interest (VOI) for measuring local quantitative values. The most commonly used method to measure a particular region is the manual region of interest (ROI)‐based approach. However, the ROI‐based approach has an inherent bias introduced by selecting individual regions of the brain, whereas automated segmentation eliminates inter‐observer variance and provides an unbiased and comprehensive assessment. The results of this study show that MRF provides reliable morphological information that could be used for automated VOI analysis, as well as to obtain regional cortical thickness and volumes.

Accurate volumetric segmentation of the brain is a prerequisite for the detection of subtle regional changes in quantitative relaxation times, which are caused by numerous diseases (Blystad et al., 2016; Dean 3rd et al., 2017; Hagiwara et al., 2019; House et al., 2006; Townsend et al., 2004; von Oertzen et al., 2002). Our results demonstrate that 3D MRF provides anatomical information that is as reliable as that provided by conventional 3D T1‐weighted imaging. Along with the highly reliable T1 and T2 value quantification, 3D MRF can identify subtle regional changes that may have been obscured during observation of large regions. The wCV of the regional changes of T1 and T2 measured by 3D MRF were highly repeatable (0.3–2.0% and by 3.6–12.9%, respectively). Morphometry and relaxometry provide complementary information, and using both may provide insights into tissue abnormalities.

We recognize several limitations associated with our study. First, we could not obtain the truly basal morphometric measurements, which is the case with other studies involving living human subjects. Objectively evaluating morphological information from MRI is difficult, which justifies the comparison of MRF‐derived measures with standard 3D T1‐weighted image‐derived thickness estimation. Although the results are dependent on the segmentation algorithm and cannot be generalized, employing commonly used automatic measurement software could be appropriate for surrogate metrics. Second, this study was performed in a healthy population. Although repeatability and reproducibility in patients may be desired for clinical trials, performing scan‐rescan tests in patients is impractical and unethical. Third, we only included a single scanner, in a single institution. Further longitudinal and multicenter studies incorporating scanners from different sites with different field strengths and manufacturers would be beneficial for establishing biomarkers with MRF that can be robustly used in clinical practice.

The repeatability reported in this study could potentially be improved using an optimized acquisition schedule, a more advanced reconstruction algorithm (Asslander et al., 2018; Bustin et al., 2019; Pierre, Ma, Chen, Badve, & Griswold, 2016) or by reducing the discretization of dictionary using a finer step size or dictionary‐free neural network based inference (Cohen, Zhu, & Rosen, 2018; Golbabaee, Chen, Gómez, Menzel, & Davies, 2019; Gómez et al., 2020; Virtue, Yu, & Lustig, 2017). Additionally, the use of multichannel inputs (e.g., T1 and T2 maps), which could be obtained from a single 3D MRF sequence scan, could potentially improve the robustness of segmentation algorithms that rely only on T1‐weighted images. This could increase robustness on the brain surface, where a large gradient in T2 relaxation time is produced by cerebrospinal fluid.

## 5 CONCLUSIONS

3D MRF‐derived measurements of human brain cortical thickness and subcortical volumes are highly repeatable and are similar to the metrics obtained using the conventional 3D T1‐weighted images. However, a slight but consistent bias exists between the two, and thus careful attention is required when combining data from MRF and conventional acquisitions.

## Supporting information


**Figure S1** Acquisition. (a) Shows the flip angle list used (880 pulses), which was preceded by an adiabatic inversion pulse and repeated for 56 acquisition segments, changing each time the rotation of spiral k‐space trajectories as shown in (b).
**Figure S2**. EPG simulation for one, two and three repetitions of the schedule (T1 = 3,000 ms and T2 = 50 ms). While at the beginning of the first repetition the magnetization is in its equilibrium state, for the second repetition the initial magnetization depends on the spin history, leading to a different signal evolution. However, for the subsequent repetitions, the initial magnetization reaches a pseudo‐equilibrium state. As a consequence, signal evolutions for repetition three (and the following) is the same as for repetition two, and it is therefore unnecessary to repeat the whole simulation more than two times to obtain the signal evolution corresponding to the acquisition.
**Figure S3.** Difference between MRF and FSPGR‐derived cortical thicknesses. (a) Bland–Altman plot showing bias between MRF and FSPGR‐derived cortical thicknesses in areas with relatively thin cortical thickness. The visual cortex areas (pericalcarine, cuneus, lingual) area are colored in red, which were consistent with the regions where the bias was observed. (b) (top) MRF‐based T1‐weighted image and FSPGR image, shown side‐by‐side. (middle) Zoomed image showing the visual cortex areas where the bias was observed between the two sequences. (bottom) Segmented labels overlaid on T1‐weighted images. Note the low gray/white matter contrast on the FSPGR images, and the missegmentation of the cortical thicknesses in visual cortex areas (pericalcarine, cuneus, lingual).
**Figure S4.** Scan‐rescan repeatability of subcortical volumes for 3D MRF and 3D FSPGR using FreeSurfer. (a) Scan‐rescan within‐subject coefficient of variation (wCV) of subcortical volumes for 3D MRF and FSPGR (Boxes indicate the interquartile range [25–75%] and circles indicate sample data points). (b) Bland–Altman plots of scan‐rescan variation in subcortical structure volumes across all subjects in all structures using (top) 3D MRF and (bottom) 3D FSPGR.
**Figure S5.** Scan‐rescan repeatability of T1 and T2 value of subcortical structures derived with 3D MRF using FreeSurfer. (a) Scan‐rescan within‐subject coefficient of variation (wCV) of 3D MRF‐derived T1 and T2 values (Boxes indicate the interquartile range [25–75%] and circles indicate sample data points). (b) Bland–Altman plots of scan‐rescan variation in 3D MRF‐derived (top) T1 and (bottom) T2 values of subcortical structures across all subjects.
**Table S1.** Intraclass correlation coefficients between MRF‐ and FSPGR‐derived cortical thickness.
**Table S2.** Intraclass correlation coefficients between MRF‐ and FSPGR‐derived subcortical structure volumes.

## Data Availability

The data that support the findings of this study are available from the corresponding author upon reasonable request.
