# Supplementary material for: Repeatability and reproducibility of human brain morphometry using three‐dimensional magnetic resonance fingerprinting
Source: Hum Brain Mapp. 2020 Oct 22;42(2):275–85. doi: 10.1002/hbm.25232 (PMC7775993; doi:10.1002/hbm.25232)
Supplement: Supplementary file 1 — Figure S1 Acquisition. (a) Shows the flip angle list used (880 pulses), which was preceded by an adiabatic inversion pulse and repeated for 56 acquisition segments, changing each time the rotation of spiral k‐space trajectories as shown in (b). Figure S2. EPG simulation for one, two and three repetitions of the schedule (T1 = 3,000 ms and T2 = 50 ms). While at the beginning of the first repetition the magnetization is in its equilibrium state, for the second repetition the initial magnetization depends on the spin history, leading to a different signal evolution. However, for the subsequent repetitions, the initial magnetization reaches a pseudo‐equilibrium state. As a consequence, signal evolutions for repetition three (and the following) is the same as for repetition two, and it is therefore unnecessary to repeat the whole simulation more than two times to obtain the signal evolution corresponding to the acquisition. Figure S3. Difference between MRF and FSPGR‐derived cortical thicknesses. (a) Bland–Altman plot showing bias between MRF and FSPGR‐derived cortical thicknesses in areas with relatively thin cortical thickness. The visual cortex areas (pericalcarine, cuneus, lingual) area are colored in red, which were consistent with the regions where the bias was observed. (b) (top) MRF‐based T1‐weighted image and FSPGR image, shown side‐by‐side. (middle) Zoomed image showing the visual cortex areas where the bias was observed between the two sequences. (bottom) Segmented labels overlaid on T1‐weighted images. Note the low gray/white matter contrast on the FSPGR images, and the missegmentation of the cortical thicknesses in visual cortex areas (pericalcarine, cuneus, lingual). Figure S4. Scan‐rescan repeatability of subcortical volumes for 3D MRF and 3D FSPGR using FreeSurfer. (a) Scan‐rescan within‐subject coefficient of variation (wCV) of subcortical volumes for 3D MRF and FSPGR (Boxes indicate the interquartile range [25–75%] and circles indicate sample data point [file HBM-42-275-s001.docx]

**Supporting information**

**Figure S1.** Acquisition. (a) Shows the flip angle list used (880 pulses), which was preceded by an adiabatic inversion pulse and repeated for 56 acquisition segments. The angle was changed with each rotation of spiral k-space trajectories, as shown in (b).


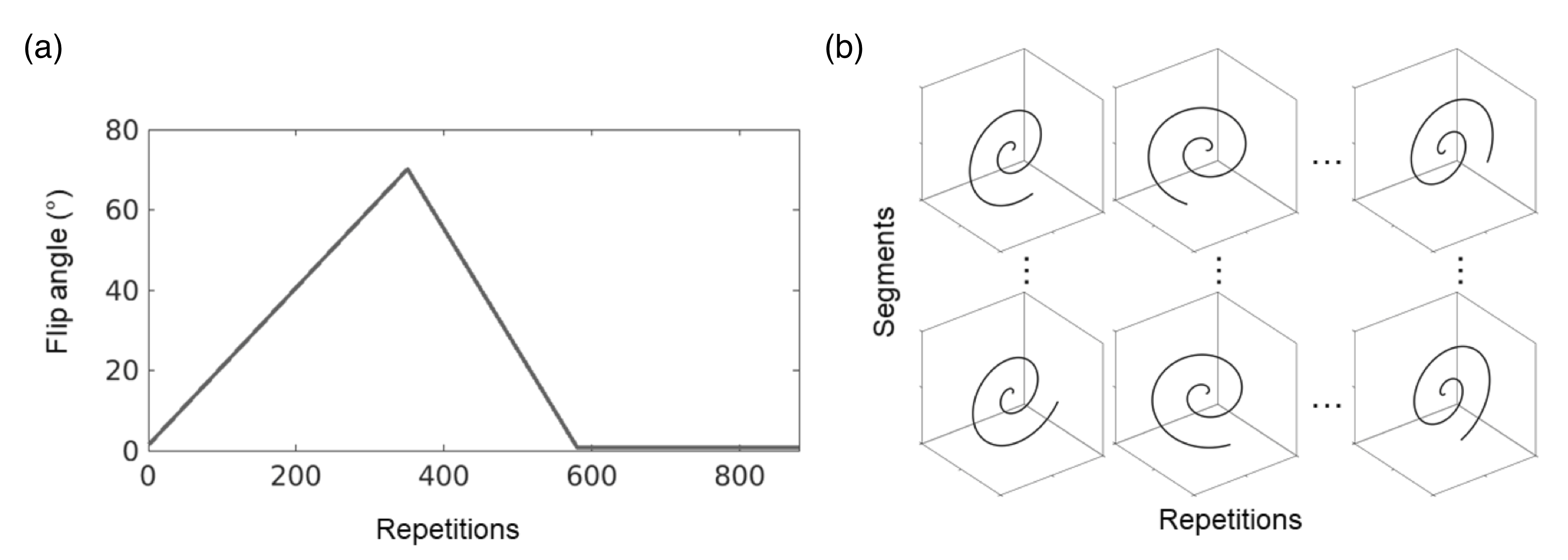


**Figure S2**. EPG simulation for one, two and three repetitions of the schedule (T1=3000 ms and T2=50 ms). While at the beginning of the first repetition the magnetization is in its equilibrium state, for the second repetition the initial magnetization depends on the spin history, leading to a different signal evolution. However, for the subsequent repetitions, the initial magnetization reaches a pseudo-equilibrium state. As a consequence, signal evolutions for repetition three (and the following) is the same as for repetition two, and it is therefore unnecessary to repeat the whole simulation more than two times to obtain the signal evolution corresponding to the acquisition.


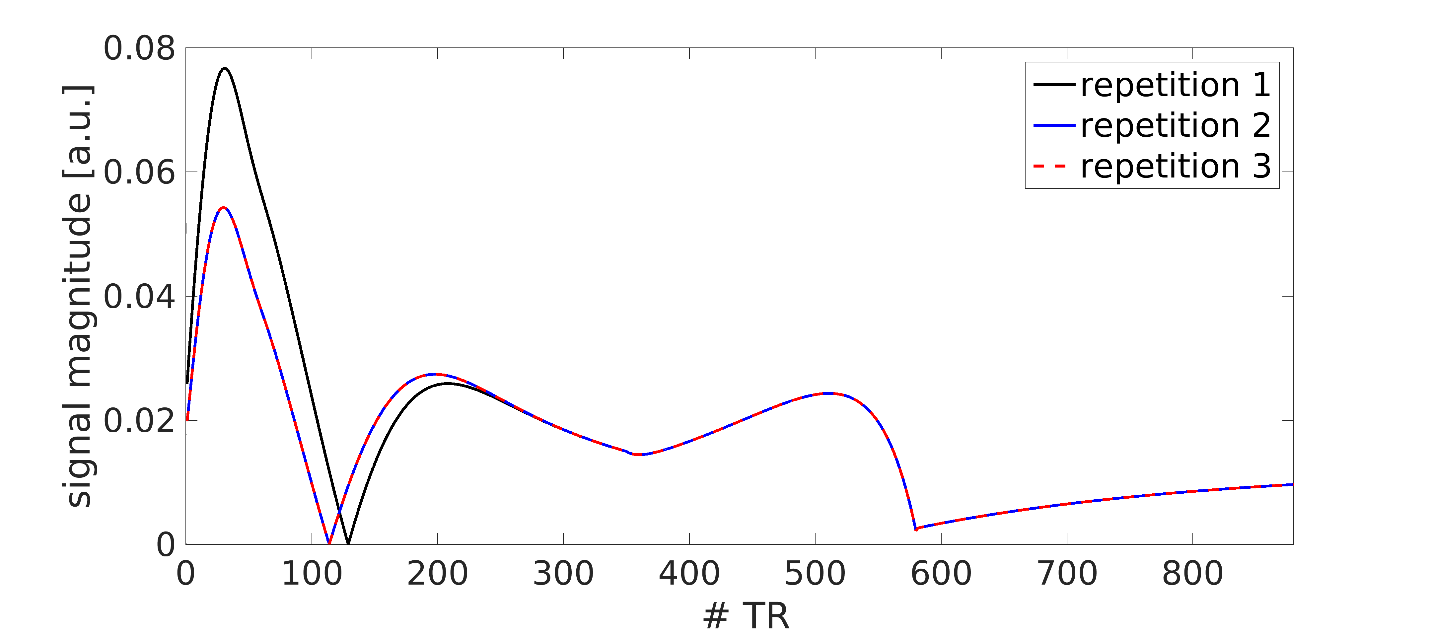


**Figure S3.** Difference between MRF and FSPGR-derived cortical thicknesses. (a) Bland–Altman plot showing bias between MRF and FSPGR-derived cortical thicknesses in areas with relatively thin cortical thickness. The visual cortex areas (pericalcarine, cuneus, lingual) area are colored in red, which were consistent with the regions where the bias was observed. (b) (top) MRF-based T1-weighted image and FSPGR image, shown side-by-side. (middle) Zoomed image showing the visual cortex areas where the bias was observed between the two sequences. (bottom) Segmented labels overlaid on T1-weighted images. Note the low gray/white matter contrast on the FSPGR images, and the missegmentation of the cortical thicknesses in visual cortex areas (pericalcarine, cuneus, lingual).


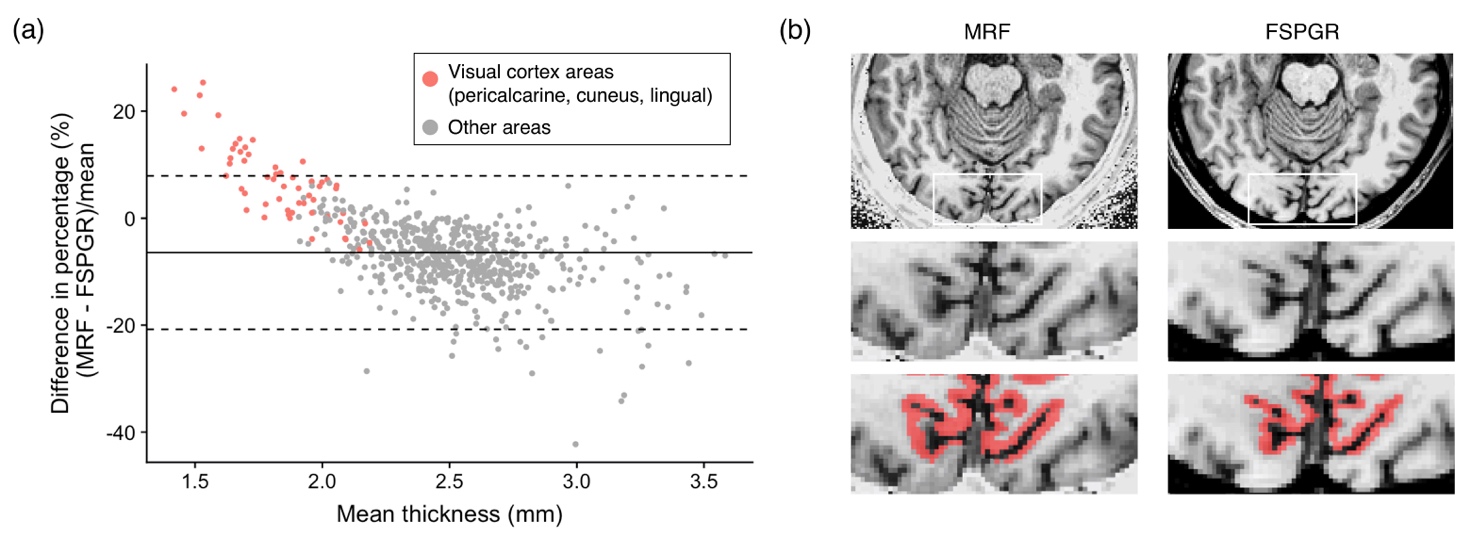


**Figure S4.** Scan-rescan repeatability of subcortical volumes for 3D MRF and 3D FSPGR using FreeSurfer. (a) Scan-rescan within-subject coefficient of variation (wCV) of subcortical volumes for 3D MRF and FSPGR (Boxes indicate the interquartile range [25–75%] and circles indicate sample data points). (b) Bland–Altman plots of scan-rescan variation in subcortical structure volumes across all subjects in all structures using (top) 3D MRF and (bottom) 3D FSPGR.


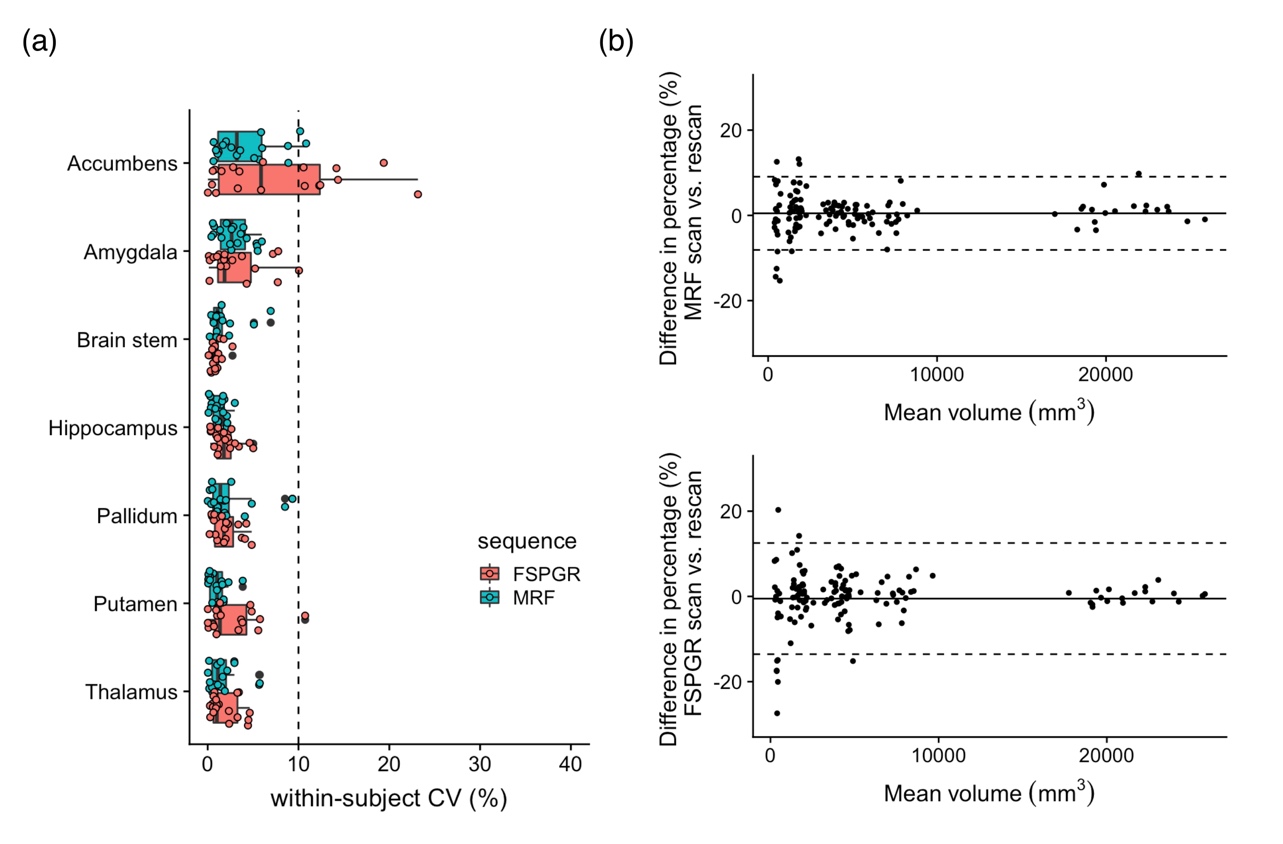


**Figure S5.** Scan-rescan repeatability of T1 and T2 value of subcortical structures derived with 3D MRF using FreeSurfer. (a) Scan-rescan within-subject coefficient of variation (wCV) of 3D MRF-derived T1 and T2 values (Boxes indicate the interquartile range [25–75%] and circles indicate sample data points). (b) Bland–Altman plots of scan-rescan variation in 3D MRF-derived (top) T1 and (bottom) T2 values of subcortical structures across all subjects.


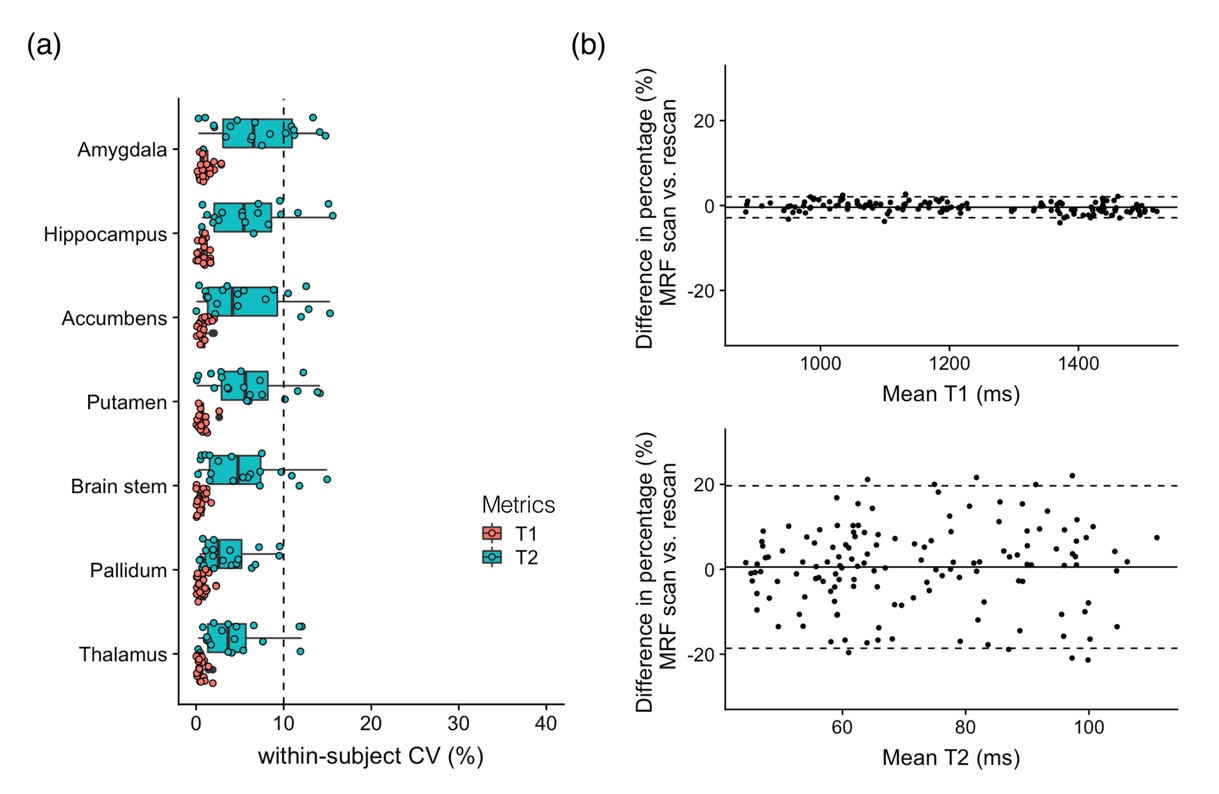


**Table S1.** Intraclass correlation coefficients between magnetic resonance fingerprinting (MRF)- and fast spoiled gradient recalled echo (FSPGR)-derived cortical thickness.

| Measurement | MRF - FSPGR | MRF  scan-rescan | FSPGR  scan-rescan |
| --- | --- | --- | --- |
| Banks of the superior temporal sulcus | 0.827 | 0.945 | 0.953 |
| Caudal anterior cingulate | 0.764 | 0.845 | 0.934 |
| Caudal middle frontal | 0.830 | 0.946 | 0.961 |
| Cuneus | 0.727 | 0.934 | 0.968 |
| Entorhinal | 0.292 | 0.824 | 0.398 |
| Fusiform | 0.304 | 0.834 | 0.923 |
| Inferior parietal | 0.507 | 0.756 | 0.949 |
| Inferior temporal | 0.444 | 0.897 | 0.915 |
| Isthmus cingulate | 0.662 | 0.861 | 0.906 |
| Lateral occipital | 0.418 | 0.476 | 0.958 |
| Lateral orbitofrontal | 0.248 | 0.704 | 0.938 |
| Lingual | 0.620 | 0.953 | 0.945 |
| Medial orbitofrontal | 0.476 | 0.553 | 0.939 |
| Middle Temporal | 0.575 | 0.937 | 0.942 |
| Para hippocampal | 0.843 | 0.936 | 0.967 |
| Para central | 0.725 | 0.819 | 0.885 |
| Pars opercularis | 0.748 | 0.878 | 0.955 |
| Pars orbitalis | 0.385 | 0.903 | 0.955 |
| Pars triangularis | 0.749 | 0.915 | 0.960 |
| Pericalcarine | 0.708 | 0.911 | 0.896 |
| Postcentral | 0.794 | 0.886 | 0.866 |
| Posterior cingulate | 0.580 | 0.877 | 0.921 |
| Precentral | 0.800 | 0.854 | 0.863 |
| Precuneus | 0.562 | 0.923 | 0.950 |
| Rostral anterior cingulate | 0.684 | 0.681 | 0.940 |
| Rostral middle frontal | 0.606 | 0.884 | 0.971 |
| Superior frontal | 0.763 | 0.883 | 0.964 |
| Superior parietal | 0.567 | 0.863 | 0.930 |
| Superior temporal | 0.798 | 0.903 | 0.966 |
| Supramarginal | 0.699 | 0.901 | 0.934 |
| Frontal pole | 0.233 | 0.639 | 0.876 |
| Temporal pole | 0.000 | 0.787 | 0.462 |
| Transverse temporal | 0.815 | 0.878 | 0.960 |
| Insula | 0.752 | 0.814 | 0.867 |

**Table S2.** Intraclass correlation coefficients between magnetic resonance fingerprinting (MRF)- and fast spoiled gradient recalled echo (FSPGR)-derived subcortical structure volumes.

| Measurement | MRF - FSPGR | MRF  scan-rescan | FSPGR  scan-rescan |
| --- | --- | --- | --- |
| Thalamus | 0.776 | 0.974 | 0.995 |
| Caudate | 0.884 | 0.765 | 0.948 |
| Putamen | 0.906 | 0.904 | 0.896 |
| Pallidum | 0.847 | 0.865 | 0.950 |
| Brain stem/4th-Ventricle | 0.913 | 0.948 | 0.952 |
| Nucleus Accumbens | 0.877 | 0.815 | 0.929 |
| Hippocampus | 0.922 | 0.948 | 0.939 |
| Amygdala | 0.581 | 0.893 | 0.835 |
